# The Rare Cases of Parotid Gland Arteriovenous Malformations

**DOI:** 10.1155/2021/6072155

**Published:** 2021-09-02

**Authors:** Manish Gupta, Vijay Shrawan Nijhawan, Cynthia Kaur, Sukhpreet Kaur, Akanksha Gupta

**Affiliations:** ^1^Department of ENT, Maharishi Markandeshwar Institute of Medical Sciences & Research MMDU, Ambala, India; ^2^Department of Pathology, Maharishi Markandeshwar Institute of Medical Sciences & Research MMDU, Ambala, India; ^3^School of Medical Science and Research, Sharda University, Greater Noida, India

## Abstract

Arteriovenous malformation (AVM) results from errors in vascular development during embryogenesis; absent capillary beds lead to shunting directly from the arterial to venous circulation. Although it is common in the head and neck region, AVMs located in the parotid gland are quite rare. Here, we report two cases of arteriovenous malformation of the parotid gland that presented to our out-patient setup with swelling in the parotid region and were diagnosed as arteriovenous malformation on histopathological study after surgical resection.

## 1. Introduction

The parotid gland is the most common site for salivary gland tumors. But, it is rare to find a vascular anomaly in the parotid gland in adults. The general terms, “Angioma” is still used for both vascular tumors and vascular malformations. Vascular tumors can be either benign or malignant, and on the other hand, vascular malformations can be capillary, venous, arteriovenous, etc.

Earlier, all vascular lesions were referred to as haemangiomas. Nowadays, they are classified into haemangiomas and vascular malformations on the basis of the endothelial characteristics. The vascular malformations are either low flow such as capillary, lymphatic, and venous malformations or high flow such as arteriovenous malformations (AVMs) [1].

We hereby present two interesting and rare cases of arteriovenous malformation of the parotid gland.

## 2. Case 1

A 32-year-old female presented to our out-patient department with complaints of swelling below the right ear for 8 years, which was insidious in onset and gradually progressive in size. It did not alter in size on chewing of food, opening and closing of mouth, neck movements, or weather changes. It was associated with a mild-to-moderate dull ache radiating to the ipsilateral side of the face.

**Figure 1:**
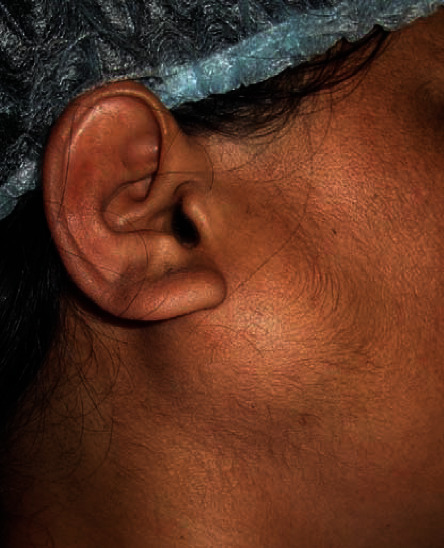
Preoperative clinical picture showing the well-defined swelling infra-auricular region.

On physical examination, the patient had a soft, compressible, nontender, nonfluctuant, nonreducible, nonpulsatile, well-defined mass in the right parotid region measuring 4 × 3 cm extending from the right lobule to 1.5 cm below the angle of the mandible in vertical dimension and 3 cm in front of the lobule in horizontal dimension (Figure 1). On auscultation, no bruit was heard.

**Figure 2:**
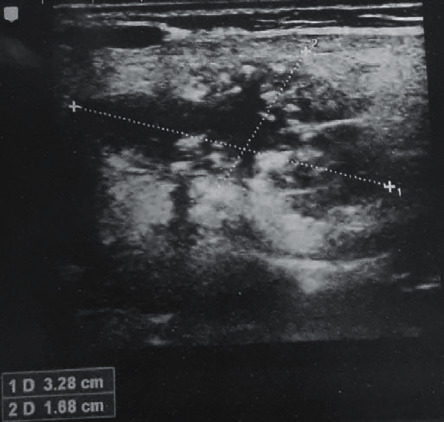
Picture showing ultrasound imaging, suggestive of arteriovenous malformation.

**Figure 3:**
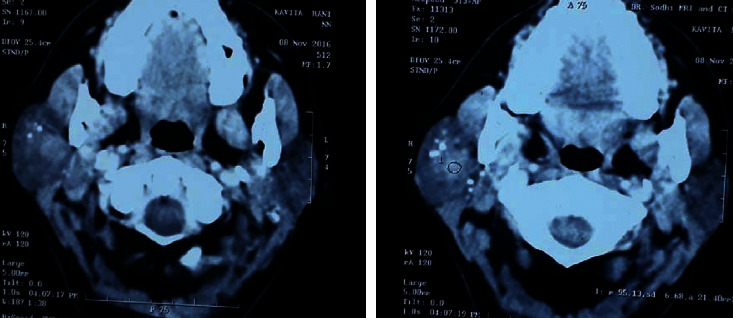
Axial cut contrast-enhanced computed tomogram showing a heterogeneously enhancing mass in the superficial lobe of the parotid gland with multiple pin head calcified foci in it.

On ultrasonography, the mass showed multiple pinhead circular calcified foci in it (Figure 2). The colour Doppler suggested an arteriovenous malformation. Computed tomography reported evidence of a heterogeneously enhancing mass lesion in the superficial lobe of the right parotid gland, and the deep lobe was normal (Figure 3).

**Figure 4:**
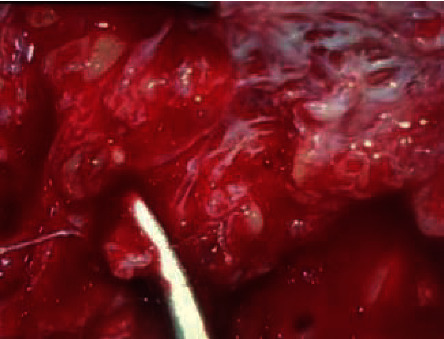
Intraoperative picture of dilated, irregular vascular channels replacing the superficial parotid parenchyma.

The patient was taken up for right superficial parotidectomy. Intraoperatively, there was diffuse, dilated vascular channels replacing parotid tissue, with retromandibular vein as the draining vessel to the mass (Figure 4). The facial nerve and its branches were identified and preserved. Postoperatively, the patient developed neuropraxia of the right facial nerve but improved within a span of 1 month on physiotherapy and oral steroids.

**Figure 5:**
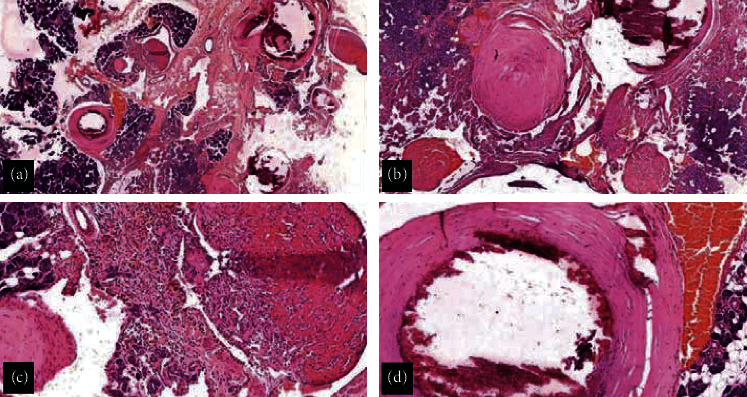
(a) Scanner photomicrograph shows thick- and thin-walled blood vessels traversing the parotid parenchyma. Thrombi and calcification are seen within the lumen (*H* and *E* x40); (b) low-power photomicrograph shows thrombi in varying stages of organisation. Marked calcification seen in one of the vessels (*H* and *E* x100); (c) some of the vascular spaces show organising thrombus with revascularization (*H* and *E* x100); and (d) low-power photomicrograph shows luminal as well as focal calcification within the wall (*H* and *E* x100).

Gross examination of the specimen showed hemorrhagic areas on the cut section. Histopathological examination conducted microscopically showed variable size vascular channels. The vessels with thick muscular walls showed hyalinisation and focal calcification. The venous calibre vessels showed intraluminal fresh thrombus, which at places showed recanalization and endothelial proliferation, consistent with A-V malformation (Figure 5).

## 3. Case 2

**Figure 6:**
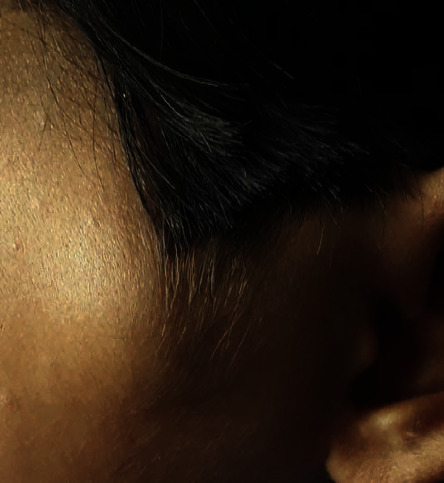
Preoperative clinical picture showing swelling in the left parotid region.

A 43-year-old male presented in ENT OPD with complaints of swelling below and in front of the left ear for 1.5 years, which was gradually progressive in nature, painless, and had alteration in size due to external factors and did not relieve with anti-inflammatory drugs. On physical examination, it was a soft, mobile, compressible, nontender, nonreducible, and nonfluctuant mass measuring around 4 × 4 cm in the left parotid region with no bruit on auscultation (Figure 6).

**Figure 7:**
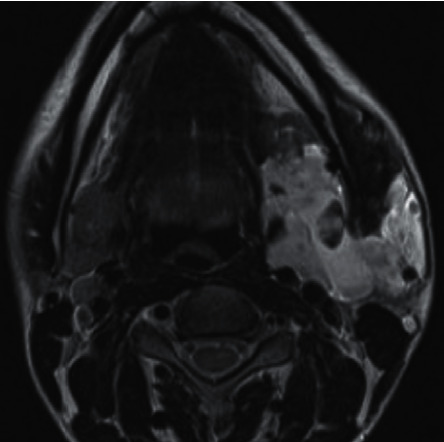
Axial-section *T*2-weighted MRI sequence showing a well-marginated lesion involving whole of the left parotid gland, hyperintense with lobulated hypointense foci within it.

Ultrasonography suggested tortuous and dilated vascular channels in the left parotid region. FNAC yielded 6 to 7 ml fresh blood and suggested benign vascular swelling. MRI showed a well-marginated mass lesion involving whole of the left parotid gland, hyperintense on the T2W sequence with few lobulated hypointense foci within it, closely abutting the left-sided retromandibular vein suggestive of arteriovenous malformation (Figure 7).

The patient was taken up for total conservative parotidectomy. The retromandibular vein was found to be the draining vessel and ligated. The facial nerve and its branches were identified and preserved. Postop period was uneventful.

**Figure 8:**
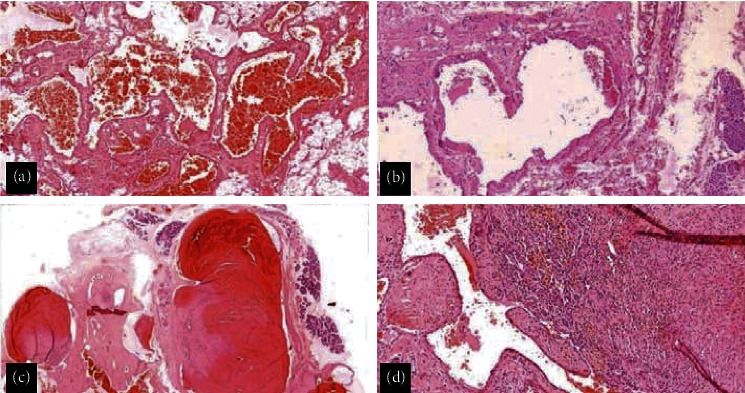
(a): Photomicrograph shows large, ectatic, thin-walled anastomosing vascular channels (*H* and *E* x40); (b) section shows a thin layer of smooth muscle within the walls of the vascular spaces with adjacent salivary acini (*H* and *E* x40); (c) salivary gland acini are seen in between the vascular spaces, with some of them showing fresh thrombi (*H* and *E* x40); and (d) organising thrombus with revascularization in a dilated vascular channel (*H* and *E* x100).

Gross examination showed tiny cystic and hemorrhagic areas on the cut section and microscopically showed fibrous-connective and mature adipose tissue showing admixture of malformed blood vessels comprising arteries and venules suggestive of arteriovenous malformation (Figure 8).

## 4. Discussion

Blood vessels have a single layer of endothelial cells, and vascular anomalies are the result of defects at the level of vasculogenesis. For the first time, the vascular lesions were classified into haemangiomas and malformation based on endothelial characteristics [2].

To understand the vascular anomalies better, the ISSVA (International Society for Study of Vascular Anomalies) recently came up with a classification system that differentiates vascular tumors (lesions with manifestations of cell proliferation and increased mitotic activity) from vascular malformations (congenital vascular anomalies due to developmental error). The latter are classified according to the type of vessel involved, i.e., capillary, venous, lymphatic, and arteriovenous malformations [1].

Another way of classifying these lesions is by their vascular flow characteristics into low-flow (such as haemangiomas, capillary, venous, and lymphatic malformations) and high-flow lesions (such as arteriovenous malformation).

High-flow malformations are further separated into arteriovenous fistulas (AVFs) and arteriovenous malformations. The fistulas are functionally open embryological residues between the arteries and veins and are present at birth. Local trauma, partial surgery, psychological stress, and endocrine event (puberty, pregnancy, and birth) may cause the fistulas to grow and form malformation [3, 4].

Vascular malformation of the parotid gland is an extremely rare condition with around 50 reported cases in the literature. A case study conducted by Beahrs et al. showed only 0.5% of the cases from 760 parotid tumours to be vascular malformations, and similarly, a study conducted by Byars et al. showed 0.6% of malformation cases from 460 reported cases of parotid tumours [5, 6]. In a more recent study conducted by Achache et al., parotid malformations accounted for only 1.6% (10 out of 614 patients) of all parotid tumour cases collected over the 10-year period from 1998 to 2008 [7].

Mostly, the superficial lobe of the parotid gland gets involved in vascular malformation, but in our second case, deep lobe involvement was seen. Also, such patients present with complaints of painless slow-growing swelling on the face in the parotid region with no other associated complaints. Clinically, turkey wattle sign may be present which is the prominence or enlargement of swelling on tilting the head to the ipsilateral side or doing the Valsalva manoeuvre. This sign is considered pathognomonic of vascular malformation, but has been seen in only 3 out of 23 previously reported cases of adult parotid vascular malformation. Basically, the turkey wattle is a red vascular structure in the neck of the male turkey that can increase in size when filled with blood [8]. This sign had not been elicited in any of our cases, probably due to thick parotid fascia.

Although clinical examination clinches the diagnosis, MRI is considered the gold standard. It shows contrast uptake and hyperintense *T*2-weighted signals with the presence of phleboliths and marked enhancement, as in our cases [3].

On ultrasound, arteriovenous malformation appears as a heterogeneous lesion with feeding vessels. Colour Doppler displays high vessel density and high systolic flow and multiple sites of arteriovenous shunting, and arterialization of all veins, i.e., pulsatile flow, is always seen in AVM [9].

FNAC (fine needle aspiration cytology) is not very beneficial in diagnosing arteriovenous malformations. Achache et al. obtained cytological samples with needle aspiration in 30% of patients and were noncontributory in all cases. They may be beneficial or be considered gold standard in diagnosing parotid tumours [7].

Angiography is useful in residual lesions after surgery to show the major vessel and embolize if necessary. The angiographic features of AVMs are dilatation and lengthening of afferent arteries with early opacification (shunting) of enlarged efferent veins.

The definitive diagnosis is made on the histopathological report. The classical appearance is benign vascular proliferation of endothelial cells in the vessel walls. The vessel lumen is mainly filled with thrombus or calcifications (phleboliths). Arteriovenous malformations have poorly defined margins with thin walls and variable-sized vessels. Direct communications between vessels are also observed. Stroma shows the presence of adipose tissue, lymphoid follicles, and smooth muscles. Immunohistochemistry (IHC) shows positivity to vascular markers such as CD31 and CD34, not carried out in our cases due to cost issues [1].

Surgical resection is the gold standard treatment, as being performed in our cases, but other options include laser, cryotherapy, embolization, and corticosteroids [1].

Embolization or sclerotherapy can also be performed prior to surgical resection to reduce the size of the lesion. It involves injecting a chemical into the AVM to shrink it and reduce the bleeding intraoperatively, but not performed in our cases to avoid damage to the facial nerve, passing through the lesion. Complications include mucosal injury, anaphylaxis, and/or nerve injury [10, 11]. Along with usage of chemicals, alcohol sclerotherapy such as ethanol has also shown tremendous results in shrinking the lesion and reducing the recurrence rate [12].

## 5. Conclusions

The world literature suggests arteriovenous malformations of the parotid gland are extremely rare in adults. The clinical examination and MRI do help in provisionally diagnosing the disease, but histopathological examination is confirmatory. Surgical resection such as superficial or total conservative parotidectomy with preservation of the facial nerve remains the mainstay treatment till date.

## Data Availability

Previously reported (case reports, case series, and review articles as mentioned in references) data were used to support this study and are available at DOI. These prior studies (and datasets) are cited at relevant places within the text as references
